# Supplementary material for: Direct observation of orbital hybridisation in a cuprate superconductor
Source: Nat Commun. 2018 Mar 6;9:972. doi: 10.1038/s41467-018-03266-0 (PMC5840306; doi:10.1038/s41467-018-03266-0)
Supplement: Supplementary file 1 — Supplementary Information [file 41467_2018_3266_MOESM1_ESM.pdf]

Direct Observation of Orbital Hybridisation in a Cuprate Superconductor

Matt et al.

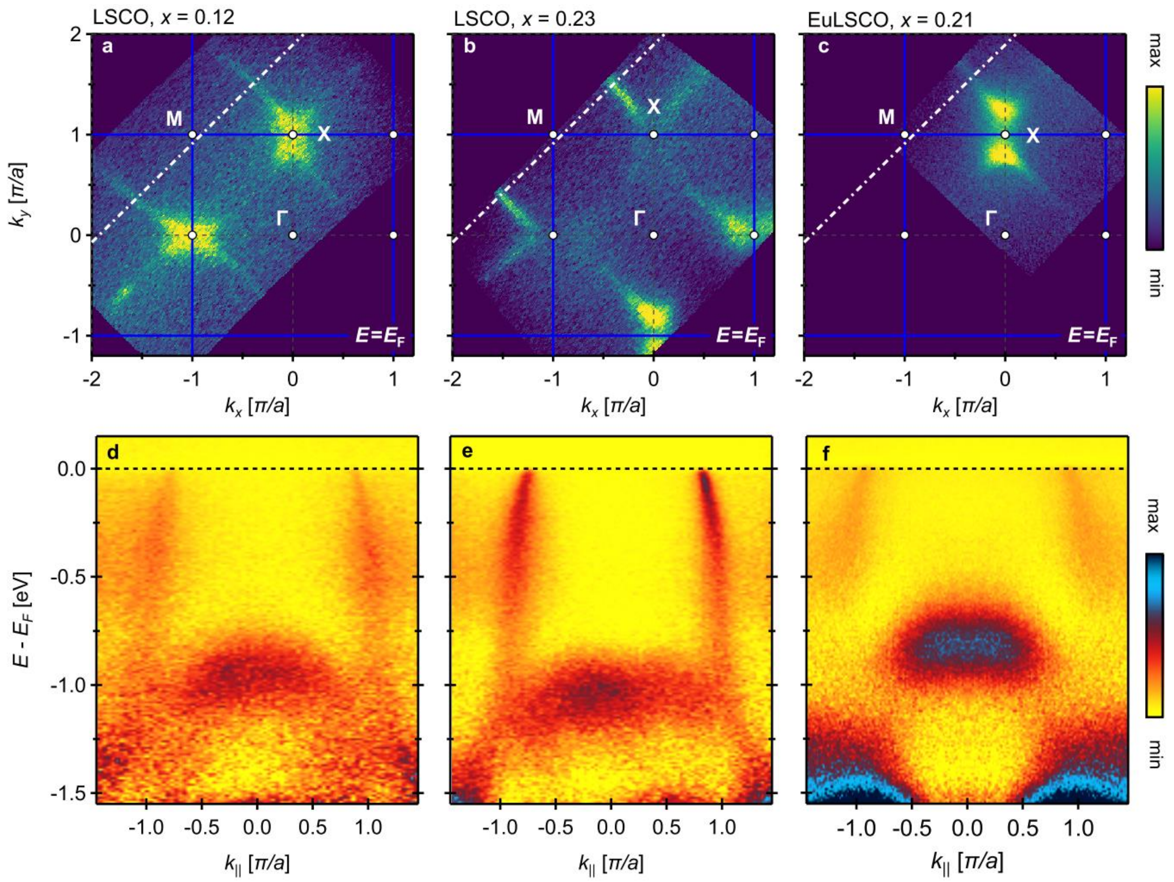

Supplementary Figure 1: Electronic band structure in $La_{2-x}Sr_xCuO_4$ (LSCO) and $Eu_{0.2}La_{1.8-x}Sr_xCuO_4$ (Eu-LSCO). (a)-(c), Fermi surface map of LSCO, x = 0.12, x = 0.23 and EuLSCO, x = 0.21 with hole-like (**a**) and electron-like (**b**)-**(c)** topology, respectively. All maps are recorded with 55 eV, $\overline{\pi}$ - polarized light. **(d)-(f)**, Background subtracted energy distribution maps (EDM) along nodal cut through the BZ corner (white dashed line) for the respective doping. Two bands are observed: The $d_{x^2-y^2}$-band which is crossing E_F and the d_{z^2}-band with its band-top around ~ 1 eV binding energy. The EDM for Eu-LSCO, x = 0.21 is symmetrised around $k_{||}$ = 0.

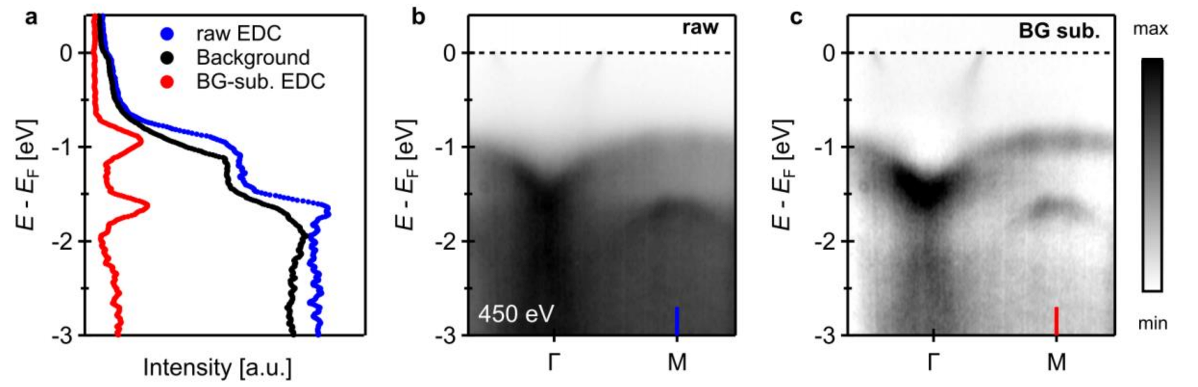

Supplementary Figure 2: Methodology for background subtraction. (a), Blue curve is an energy distribution curve (EDC) recorded on $La_{1.77}Sr_{0.23}CuO_4$ at the M-point as indicated by blue line in **b**. Black line indicates background EDC constructed by averaging the five lowest MDC-intensity points for every binding energy in the spectrum. Red points represent the background subtracted EDC at the M-point. **(b)-(c)**, Raw and background subtracted energy distribution maps along a nodal M-Γ-M direction.

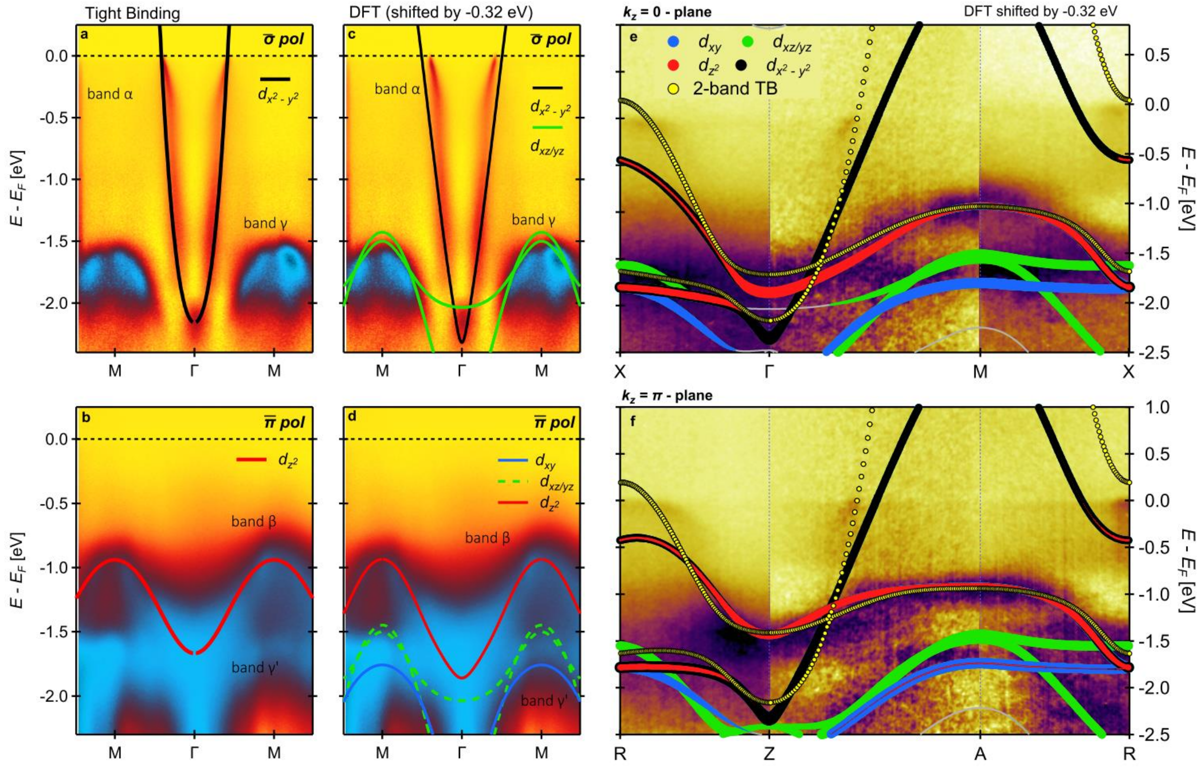

Supplementary Figure 3: Orbital character of band structure along high symmetry directions. (a) - **(b)** Two orbital tight-binding band dispersion overlaid on energy distribution maps recorded with 160 eV, σ̅ and π̅ polarised light, respectively. **(c)** - **(d)** Orbital resolved DFT band dispersion overlaid on same EDMs. The DFT bands have been downward shifted by 320 meV to fit top of d_{z^2} band. **(e)** - **(f)** Two band tight binding band dispersion and DFT band structure overlaid on EDMs obtained along high symmetry directions with 570 eV **(e)** and 525 eV **(f)**, circular polarised light, respectively.

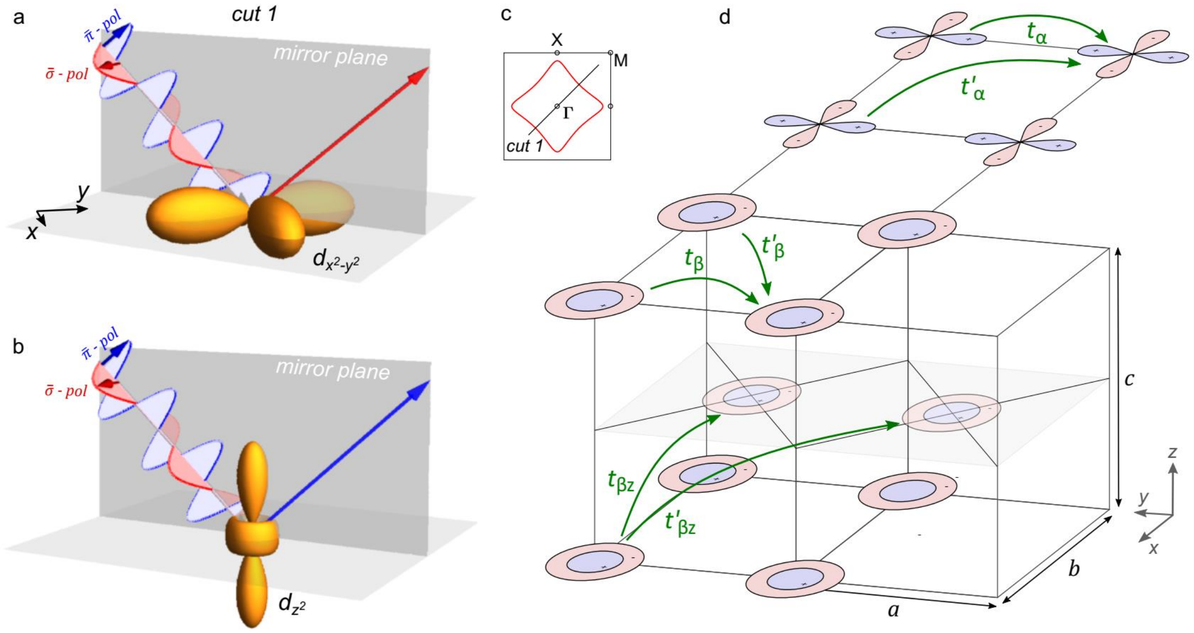

Supplementary Figure 4: Orbital character and two-band tight binding hopping parameters. (a)-(b), Schematics illustrating the mirror planes (dark grey). They are defined by the incoming light and the electron analyser slit. The electromagnetic field vector of the incoming light is situated either perpendicular to ($\bar{\sigma}$-polarised, indicated in red) or within the mirror plane ($\bar{\pi}$-polarised, indicated by blue lines). The colour of the outgoing arrow indicates the light-polarisation which has a finite photoemission cross section. (**a**) Experimental geometry illustrated with the $d_{x^2-y^2}$-orbital. (**a**) Schematics of the photoemission process on the d_{z^2} orbital. In this experimental setup, the orbitals are probed individually by switching the polarisation of the light. **(c)**, Sketch of the FS and projected Brillouin zone (BZ) for overdoped LSCO with the direction of *cut 1* as indicated. **(d)**, Schematics of crystal structure of LSCO where only Cu sites are shown by schematic drawings of $d_{x^2-y^2}$ and d_{z^2} orbitals. In-plane hopping parameters between $d_{x^2-y^2}$ (d_{z^2}) orbitals are indicated by t_α and t'_α (t_β and t'_β) while out-of-plane hopping integrals between d_{z^2} orbitals are indicated by $t_{\beta z}$ and $t'_{\beta z}$.

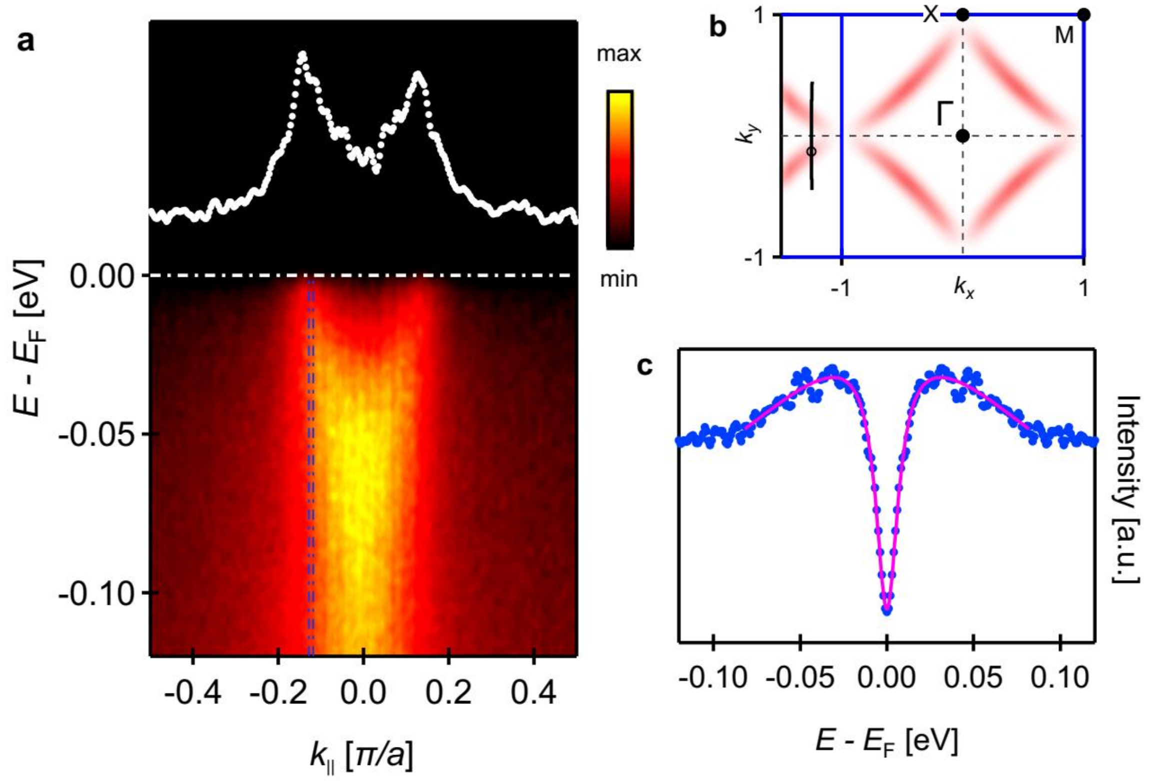

Supplementary Figure 5: Pseudogap in $La_{1.59}Eu_{0.2}Sr_{0.21}CuO_4$. (a), Anti nodal energy distribution map (EDM) recorded at ~ 20 K along direction as indicated in **b**. White dots on top show momentum distribution curve at the Fermi energy E_F. **(b),** Sketch of the Fermi surface of EuLSCO, x = 0.21. Black line indicates position of EDM in **a**. **(c),** Symmetrised energy distribution curve (EDC) at k_F as indicated by blue dashed line in **a** and circle in **b**. A pseudogap of ~ 0.03 eV is observed.

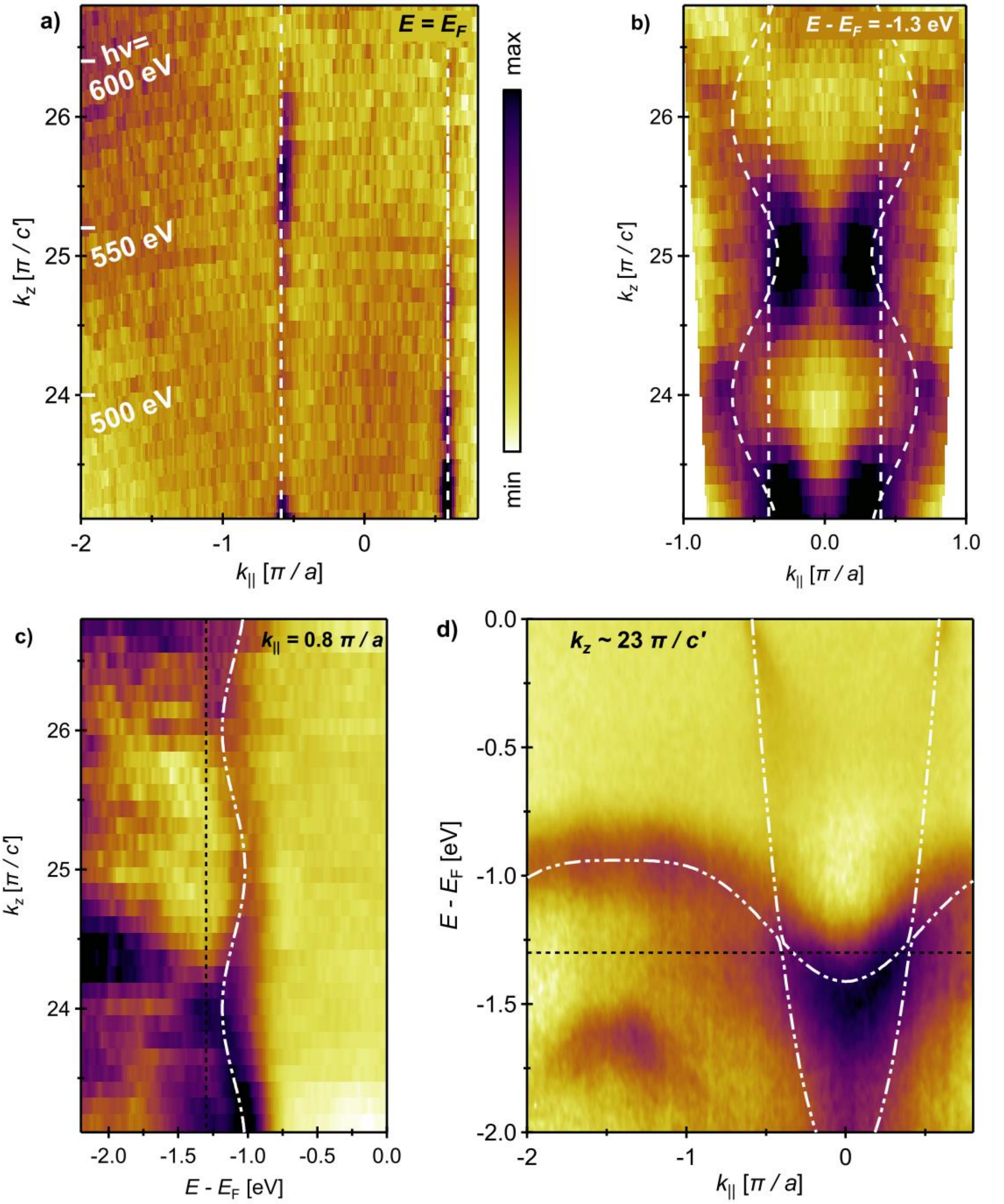

Supplementary Figure 6: Three-dimensional band dispersion of $La_{1.77}Sr_{0.23}CuO_4$. (a) - (b), Nodal k_z dispersion at E_F and at 1.3eV binding energy. **(c)** - **(d)** Energy distribution map EDM at $k_{||}$ = 0.8 π/a along the nodal (π, π) direction and at at k_z = 23 π/c'. Black dotted line represents position of constant energy map plotted in **b**. White dashed lines in all panels represent tight binding model. All spectra are recorded at T = 10K.
